# Advanced Polymeric Nanocomposites for Water Treatment Applications: A Holistic Perspective

**DOI:** 10.3390/polym14122462

**Published:** 2022-06-16

**Authors:** Adedapo Oluwasanu Adeola, Philiswa Nosizo Nomngongo

**Affiliations:** 1 Department of Chemical Sciences, Adekunle Ajasin University, Akungba Akoko 001, Ondo State, Nigeria; 2 Department of Chemical Sciences, Doornfontein Campus, University of Johannesburg, Doornfontein, Johannesburg 2028, South Africa; 3 Department of Science and Innovation-National Research Foundation South African Research Chair Initiative (DSI-NRF SARChI) in Nanotechnology for Water, University of Johannesburg, Doornfontein, Johannesburg 2028, South Africa

**Keywords:** fabrication techniques, inorganic contaminants, organic pollutants, polymer nanocomposites, water treatment

## Abstract

Water pollution remains one of the greatest challenges in the modern era, and water treatment strategies have continually been improved to meet the increasing demand for safe water. In the last few decades, tremendous research has been carried out toward developing selective and efficient polymeric adsorbents and membranes. However, developing non-toxic, biocompatible, cost-effective, and efficient polymeric nanocomposites is still being explored. In polymer nanocomposites, nanofillers and/or nanoparticles are dispersed in polymeric matrices such as dendrimer, cellulose, resins, etc., to improve their mechanical, thermophysical, and physicochemical properties. Several techniques can be used to develop polymer nanocomposites, and the most prevalent methods include mixing, melt-mixing, in-situ polymerization, electrospinning, and selective laser sintering techniques. Emerging technologies for polymer nanocomposite development include selective laser sintering and microwave-assisted techniques, proffering solutions to aggregation challenges and other morphological defects. Available and emerging techniques aim to produce efficient, durable, and cost-effective polymer nanocomposites with uniform dispersion and minimal defects. Polymer nanocomposites are utilized as filtering membranes and adsorbents to remove chemical contaminants from aqueous media. This study covers the synthesis and usage of various polymeric nanocomposites in water treatment, as well as the major criteria that influence their performance, and highlights challenges and considerations for future research.

## 1. Introduction

Water plays a major role in the evolution of human civilization and industrialization. The population of the world is predicted to increase to 9 billion people, which will result in an increase in demand for freshwater and necessitate wastewater treatment and reuse [1,2,3]. In recent decades, human activities have exacerbated major environmental and conservation challenges [4,5,6]. The environmental challenges we currently face that pose a threat to sustainable life on land (15th Sustainable Development Goal) include water and air pollution, poor waste management, fallen groundwater tables, loss of biodiversity, land/soil debasement, global warming/climate change, depletion of natural resources, etc. [7,8,9].

Nanomaterials could be a viable and effective technique for overcoming significant obstacles in the development of efficient remedial technologies and environmental protection [10,11]. However, within the last century, the volume of industrial chemicals produced has increased dramatically, from 1 to 400 million tons in the year 2000 [12]. Furthermore, between 2000 and 2017, the worldwide production capacity of industries increased from 1.2 billion tons to 2.3 billion tons [13]. The advances in science and the phenomenal growth in production capacity in the 21st century have also resulted in the emergence of troubling realities, such as the exponential increase in the spectrum of different classes of emerging pollutants detected in our water systems. As a result, new materials are urgently needed to clean up the polluted environment.

Nanostructured sorbents have a high capacity for the treatment of polluted water and may be tailored to target specific pollutants [10,11]. There have been recent improvements in the development of polymer nanocomposites (PNC), which have enhanced their novel applications in pollution remediation. Many chemical pollutants, such as dyes, heavy metals, and hydrocarbons, have been removed from wastewater using polymer nanocomposites [14,15]. Natural and synthetic polymers are both accessible. Natural polymers are those that are found in nature and can be extracted for usage. Water-based natural polymers include silk, wool, DNA, cellulose, and proteins [16]. Synthetic polymers, on the other hand, include nylon, polyethylene, polyester, Teflon, and epoxy, etc. Polymer nanocomposites possess unique synergistic features that are not possible to achieve with individual components functioning alone [17]. Inorganic nanofillers such as nanoclays, metal-oxide nanoparticles, carbon nanomaterials, and metal nanoparticles, can be introduced into a polymer matrix to create a PNC with better properties for a specific application [18,19]. Polymer nanocomposites have gained scientific prominence due to their broad range of applications in environmental remediation and treating various environmental challenges [16,20].

The aim of this review is to comprehensively examine the current state of polymeric nanocomposites as water remediation materials around the world, discussing various classifications of polymer nanocomposites, state-of-the-art synthetic methods, applications, merits, limitations, and potentials. The search keywords for this review are polymeric material, polymer nanocomposite, adsorbent, photocatalyst, organic pollutant, heavy metal, photocatalytic degradation, adsorption, and water treatment, and the literature scope is based on SCOPUS and Web of Science published papers and books.

## 2. Synthetic Methods and Remedial Application of Polymeric Nanocomposites

The choice of design, precursors, and synthetic methods for polymer nanocomposite development are germane. It entails choosing monomers, fillers, and other composite materials, as well as synthesis methods from a vast variety in order to produce PNC with the required property [21]. The determination of optimal processing strategy, considering the application intended for the PNC, and lastly, fabricating the product yield of the composite. This emphasizes the significance of the design and synthesis steps in the manufacture of PNC [18]. The ultrasonication-assisted mixing, shear mixing, microwave-assisted synthesis, roll milling, in-situ polymerization, ball milling, selective laser sintering, double-screw extrusion, and 3D printing (additive manufacturing) are among the most often utilized synthesis procedures [18,22]. Generally, there are two methods for making polymer nanocomposites: direct compounding and in situ synthesis [23]. The choice of synthetic methods demonstrates researchers’ trend toward simple, scalable, and ultimately commercially viable and reproducible PNC [24].

Atomic layer deposition and plasma-assisted mechanochemistry have been reported to solve nanoparticle aggregation challenges in melt-mixing techniques. In addition, high-frequency sonication plays a similar role in the disaggregation of nanoparticles in mixing procedures. In-situ polymerization allows for the creation of thermodynamically stable nanocomposites, while electrospinning is an efficient way of creating porous objects. Furthermore, fabricating nanocomposites using selective laser sintering offers obvious advantages in terms of overcoming the aggregation problem [25]. Due to the unique features and various suitable applications of nanofillers, they are increasingly being used in the fabrication of PNC. One-dimensional (1D) nanofillers are fillers with one dimension smaller than 100 nanometers [18]. They normally come in sheets ranging in thickness from a few nanometers to hundreds of thousands of nanometers in length. One-dimensional nanofillers include montmorillonite clay and graphene nano-platelets. Both academics and industry are interested in polymer nanocomposites made from different nanofillers and polymers [2]. Due to its consistent volumetric heating and the huge increase in reaction rate, microwave-assisted synthesis is an emerging technology used in numerous domains of materials science and chemistry. Microwave-assisted fabrication has also been used to create functionalized polymer-based nanoadsorbents [26].

### 2.1. Dendritic Polymers

**Figure 1:**
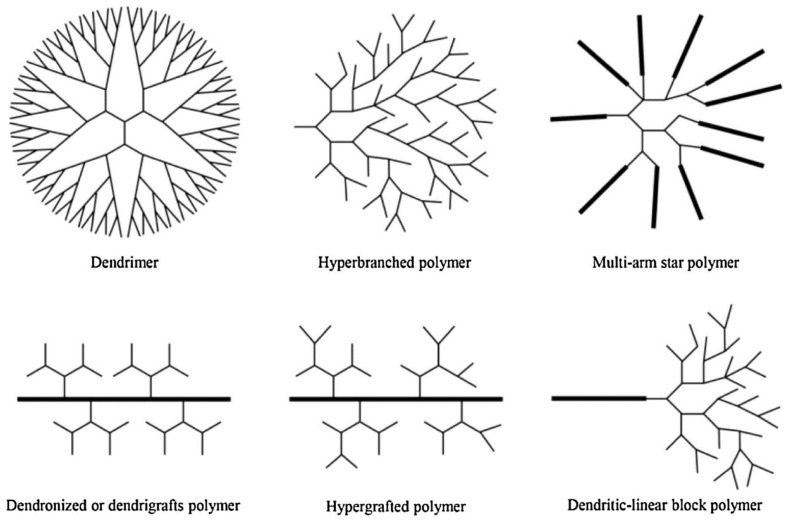
Six subclasses of dendritic polymeric material. Adapted with permission from Ma et al. [28]. Copyright (2016) Ivyspring International Publisher.

Dendrimers are branched structures with a specific form, size, and molecular weight [27,28]. Dendrimers, which are made up of monomers that radiate from a central core, are becoming a popular type of polymer. Different classes of dendritic polymers are depicted in Figure 1. Due to the large number of functional groups in the core, on the surface, and the periphery and pocket, dendrimers are a high-capacity nanoscaled multidentate chelating agent/ligand, suitable for ion separation technology. The micro-environment within the dendrimer scaffold, on the other hand, may be an appropriate host for diverse contaminants. Dendrimers’ potential for environmental clean-up has also been explored. Dendrimers’ value for removing inorganic contaminants as well as organic pollutants has been extensively demonstrated in this regard [29]. Dendrimers are also attractive chemicals for the preparation of electrochemical sensors [30].

**Figure 2:**
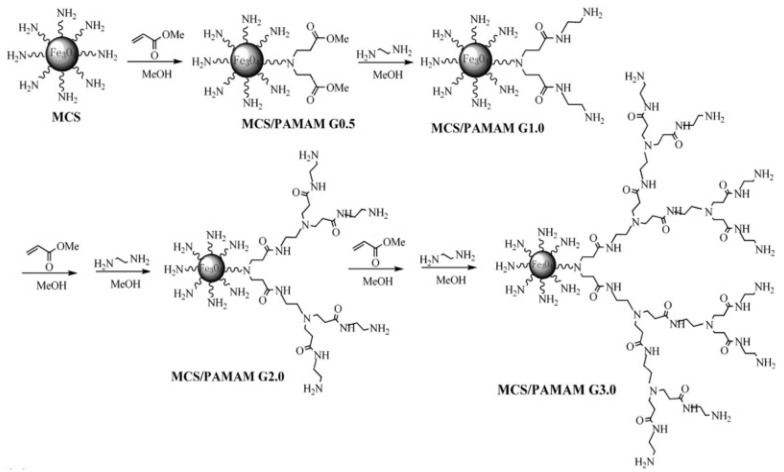
PAMAM growth on magnetic chitosan nanoparticles. Adapted with minor modifications with permission from Wang et al. [32]. Copyright (2015) Elsevier.

Poly(amidoamine) (PAMAM) dendrimers are extensively utilized dendrimers for the adsorption of pollutants in aqueous media. They are made up of ethylenediamine core, repeating units, and terminal units that make up the three fundamental units [29]. Their synthesis is performed by serially repeating two reactions: amino group addition to the double bond of methyl acrylate, accompanied by amidation of the resulting methyl ester with ethylenediamine. Each reaction step results in the production of a new dendrimer (Figure 2). The addition-amidation reaction increases the diameter of PAMAM dendrimers by increasing the repeating units. This is a 1 nm per generation increase [29,31,32].

**Figure 3:**
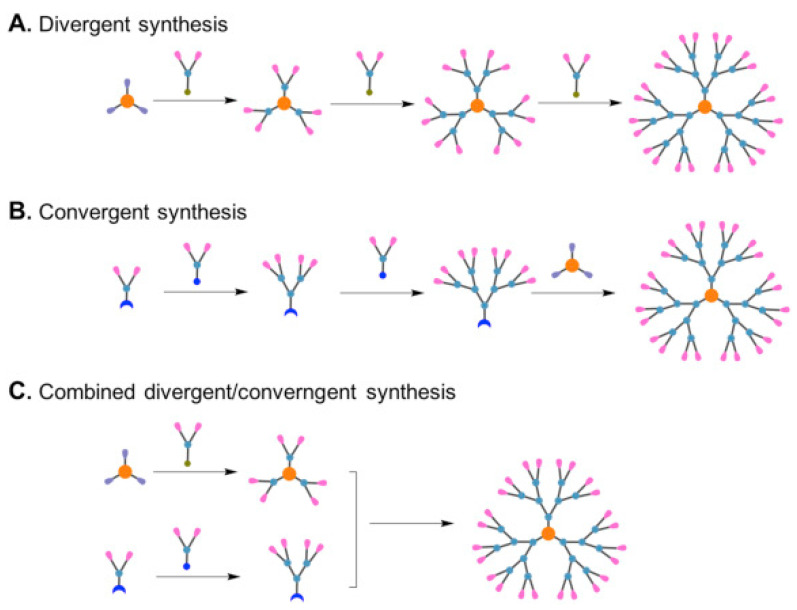
Covalent dendrimer synthesis using: (**A**) divergent synthetic route, (**B**) divergent synthetic route, (**C**) Combined approach (Adapted with permission from Lyu et al. [31]. Copyright (2019) Elsevier).

Most polymers are synthesized via single step; however, dendrimers are made in a series of steps, giving them well-tuned structures and narrow polydispersity. Divergent or convergent techniques, or a combination of both, can be used to synthesize dendrimers [33,34]. The divergent strategy entails the preparation of the dendrimer from a multifunctional core that is stretched outward by repeated reaction sequence (Figure 3A). On the other hand, a convergent method involves a bottom-up approach where dendrimers are constructed with tiny molecules that build-up on dendrimer surface and get connected to a central core through a sequence of inward-oriented interactions (Figure 3B). To combine the advantages of divergent and convergent synthesis, a combined divergent/convergent strategy, also known as the double-stage convergent approach (Figure 3C), has been further developed and employed for dendrimer synthesis [31,35].

However, structural flaws are a major problem in the synthesis of high-generation dendrimers because they are often caused by incomplete reactions or side reactions that take place as steric hindrance increases. Furthermore, due to their comparable chemical compositions and physical properties, dendrimers with structural flaws are frequently difficult to distinguish from intact dendrimers [33]. The convergent technique gives better control of the preparation of dendritic polymers, thus limiting structural flaws and impurities can be gotten rid of easily, as they conspicuously differ in morphology from the synthesized dendrimers. However, steric congestion is a challenge in the convergent approach, thus they are only suitable for small-scale dendrimers [36,37,38]. In the combination approach, a divergent technique is used to synthesize building blocks, which is then followed by convergent dendrimer assembly. Furthermore, when compared to either divergent or convergent synthesis, the sequence of reactions necessary for dendrimer preparation and purification can be minimized, thus ensuring more efficient production of higher generation [31,39].

#### Environmental Remediation Using Dendritic Polymers

Researchers are interested in dendritic polymer-based nanocomposites because of their tunable architectures and characteristics. Using appropriate functional moieties, dendritic polymers can be tailored to the target contaminants (Table 1). Dendritic PNC has high specific surface areas and pore volumes for trapping chemical contaminants. They can be combined with a variety of supports and other materials. A challenge to field/large scale applications is the multi-step synthesis, which requires profound expertise in polymer development. This challenge is solvable by collaborative research between organic chemists and separation science professionals.

Dendrimers have shown good potential for remediation of inorganic and organic pollutants (Table 1) in aqueous matrices based on the following reasons:

- They are a unique sort of macromolecules with a highly branched structure, high porosity, and a three-dimensional functionalized structure.
- Dendrimers can be grafted onto large supports, leading to increased selectivity through size exclusion by modified cavities and/or via selective binding to contaminants due to well-tailored support/substrate. Large support may also enhance surface area of the nanocomposite, leading to higher adsorption/separation capacity.
- They have large external and interior regions, as well as a large network of peripheral functional moieties, which permits the capture of large amount of contaminants.
- Adjustment of the physicochemical parameters of the core, interior cells, and outer end groups plays a major role in their adsorption capacity.
- The existence of a high number of required peripheral functional groups ensures good selectivity. More intriguingly, the character of functional groupings of the nanocomposite can be tailored to target pollutants.

Heavy metal decontamination of polluted water with aid of dendritic polymeric materials is influenced by pH due to its impacts on the moieties on the surface of dendritic polymer. Furthermore, solution pH plays a crucial role in the adsorption performance of dendritic polymeric materials by altering the speciation and shape of metal ions [29,40]. The mercury (Hg) adsorption onto silica gel-supported salicylaldehyde-modified PAMAM dendrimers was investigated using the density functional theory (DFT) approach in another study [41]. Hg ions may bind with dendritic polymers by chelation due to the presence of oxygen and nitrogen species. The results of the Dubinin–Radushkevich (D–R) isotherm model indicate that mercury was removed via chemisorption onto sorbent pores, while thermodynamic tests revealed the endothermic and spontaneous nature of the sorption process, similar to what was reported in a recent study [42].

There are hundreds of polycyclic aromatic hydrocarbons (PAHs), which are essentially hydrocarbons containing two or more fused aromatic rings. Partial combustion or burning of carbon-based materials generates hazardous PAHs [43]. They can cause endocrine disruption and are carcinogenic [44]. Arkas et al. used hyperbranched poly(ethylene imine) in combination with silicic acid to remove pyrene and phenanthrene from an aqueous solution [45]. Sol–gel processes were used to make the dendrimer–silica nanoparticle composite. It was discovered that adding dendrimer to the silica nanospheres increased the adsorption of PAHs. The development of complexes with transferable charges between the tertiary amino groups of the dendrimer and PAHs resulted in a greater water treatment performance of PEI–silica nanoparticles.

Textile dyes, particularly acid dyes, are frequently utilized and they are poisonous, causing nausea, sleepiness, diarrhea, blood clots, and breathing difficulties [37,46,47]. Textile production is critical to commerce and industry. Unexhausted dye is being thrown into natural streams without being adequately treated by some businesses due to non-compliance with environmental requirements and wastewater treatment before discharge [24]. Various colors have been removed from aqueous solutions using dendritic polymers (Table 1). Hydrogen bonding, Vander Waals forces, electrostatic attractions, and dye trapping in dendrimers facilitate dye adsorption on dendrimer-based hybrid complexes [48]. A dendritic polymer was used by Hayati et al. to remove Acid Blue 7 (AB7), Direct Red 23 (DR23), Acid Green 25 (AG25), and Direct Red 80 (DR80) aqueous solution [49]. The adsorption process was discovered to be affected by pH, dye concentration and adsorbent dosage. The Langmuir (monolayer) adsorption isotherm model was shown to be the best fit for all dye adsorption by PPI dendrimers. The dye removal rate decreases as the pH rise, with the highest adsorption capacity occurring at pH 2 as a result of substantial electrostatic interaction between the anionic dye and the positively charged dendrimer surface. Removal of dye anions reduces when pH rises as a result of the decline in the number of positively charged sites.

Dendrimers enhance the activity of the photocatalysts for dye degradation when used as supports. PAMAM dendrimer, for example, was used to modify the photocatalytic activity of a polyoxometalate (POM) cluster for methyl red (MR) degradation by blocking POM-MR aggregation. After 20 min of irradiation under the same reaction conditions, POM-dendrimer showed an increased dye degradation of 83% compared to 11% for POM [48,50]. Dendritic polymers and composites have demonstrated extraordinary potential in the field of water remediation. Dendrimers can be utilized on their own or with other materials. In dynamic systems, they can be immobilized over membranes. Dendrimer production, solution pH, contact time, and other factors influence their performance [37,51]. They are less toxic and biocompatible, which bodes well for their future commercialization and applications in water treatment.

**Table 1:** Various dendritic polymers used for the treatment of contaminated water.

Dendritic Nanocomposites	Target Pollutant	Remediation Approach	Removal Capacity	References
PAMAM/Graphene oxide	***Heavy metals***: Pb, Cd, Cu, MnCd, Cu, Mn	Adsorption	568.18, 253.81, 68.68, 18.29 253.81, 68.68, 18.29 (mg/g)	[52,53]
Dendrimer-clay nanocomposite	Cr	Adsorption	6–10 (mg/g)	[54]
Polystyrene PAMAMiminodiacetic acid	Ni	Adsorption	24.09 (mg/g)	[55]
PAMAM-grafted cellulosenanofibril	Cr	Adsorption	377.36 (mg/g)	[56]
Hyperbranched PAMAM/polysulfone membrane	Cd	Ultrafiltration	27.29 µg/cm^2^	[57]
Dendrimer/titania	Pb	Adsorption	400 (mg/g)	[58]
PAMAM-grafted core-shellmagnetic silica nanoparticles	Hg	Adsorption	134.6 (mg/g)	[59]
PAMAM dendrimers withethylenediamine (EDA) core	Cu	Ultrafiltration	451 (mg/g)	[60]
Amine terminated-Magneticcored dendrimer	Pb, Cd	Adsorption	170.42, 75.15 (mg/g)	[61]
Carbon nanotube-dendrimer	Pb, Cu	Adsorption	3333–4320 (mg/g)	[62]
Polyacrylonitrile/PAMAM composite nanofibers,	***Dyes***: Direct red 80, Direct red 23	Adsorption	2000 (mg/g)	[63]
Magnetic Chitosan/PAMAM	Reactive blue 21	Adsorption	555.56 (mg/g)	[32]
PPI–grafted cotton fabrics	Direct red 80, Disperse yellow 42, Basic blue 9	Adsorption	143.3, 104.8, 105.8 (mg/g)	[64]
PPI dendrimer	Direct red 80, Acid green 25, Acid blue 7, Direct red 23	Adsorption	33,333–50,000 (mg/g)	[49]
Graphene oxide-PPI dendrimer	Acid red 14, Acid blue 92	Adsorption	434.78, 196.08 (mg/g)	[65]
PAMAM–titaniananohybrid	Phenol	Adsorption	77 (mg/g)	[61]
PPI dendrimers functionalized with long aliphatic chains	***PAHs***: Fluoranthene, Phenanthrene, Pyrene	Adsorption	19, 67, 57 (mg/g)	[66]
Alkylated hyperbranched polymers	Fluoranthene, Phenanthrene, Pyrene	Adsorption	6–54 (mg/g)	[45]

### 2.2. Polymeric Aerogels and Hydrogels

**Figure 4:**
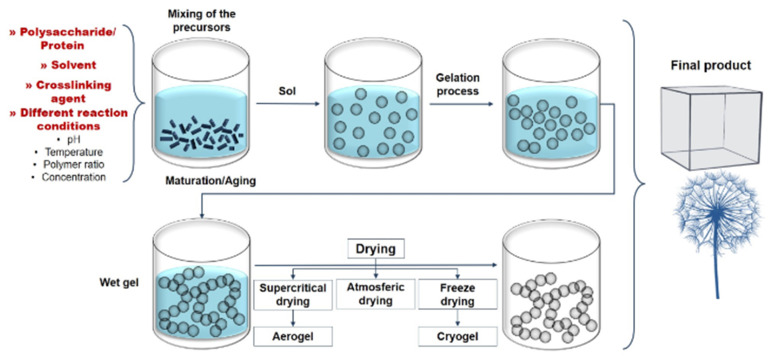
A general method for the preparation of aerogels. Adapted from Nita et al. [71].

Polymeric aerogels are unique types of porous material with interesting physicochemical features, including ultra-low thermal conductivity, low density, high porosity, large specific surface area, and controllable surface chemistry [67]. Aerogels are polymeric nanoparticle networks that are expanded by a gas across their whole volume, while hydrogels are cross-linked polymeric networks that may contain water within the interstitial spaces between chains [68,69]. The feasibility of this porous material in various applications has been thoroughly studied, thanks to recent breakthroughs in the synthesis of several forms of aerogels [70]. Aerogels and hydrogels are used as adsorbents for removing a variety of pollutants that are hazardous to the environment and human health. Figure 4 describes the general approach for the synthesis of aerogels, which involves the mixing of starting materials, gelling and a crucial step of removing solvents from pores of wet gels without distortion of the structure or network of molecules [71]. The ability to regulate the construction of a tunable aerogel network is enabled by the flexibility of the process conditions (change of the synthesis parameters, composition, etc.) [72]. The drying stage is critical for retaining porosity and integrity. The supercritical drying approach is the best method so far for achieving well-defined structures among the many drying processes, although freeze-drying is considerably easier, less expensive, and ecofriendly [73,74,75].

**Figure 5:**
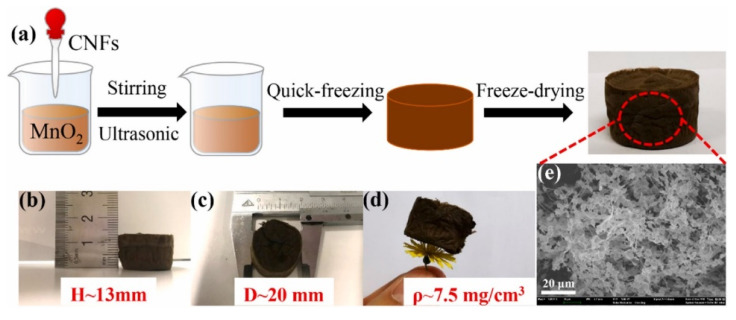
Preparation of macroporous MnO_2_-based aerogel crosslinked with cellulose nanofibers. (**a**) scheme for the synthesis of cellulose nanofibers/MnO_2_ hybrid aerogels; (**b**–**d**) the height, diameter and density of cellulose nanofibers/MnO_2_ hybrid aerogel; (**e**) the SEM image of cellulose nanofibers/MnO_2_ hybrid aerogel. Adapted with permission from Cao et al. [75]. Copyright (2021) Elsevier.

**Figure 6:**
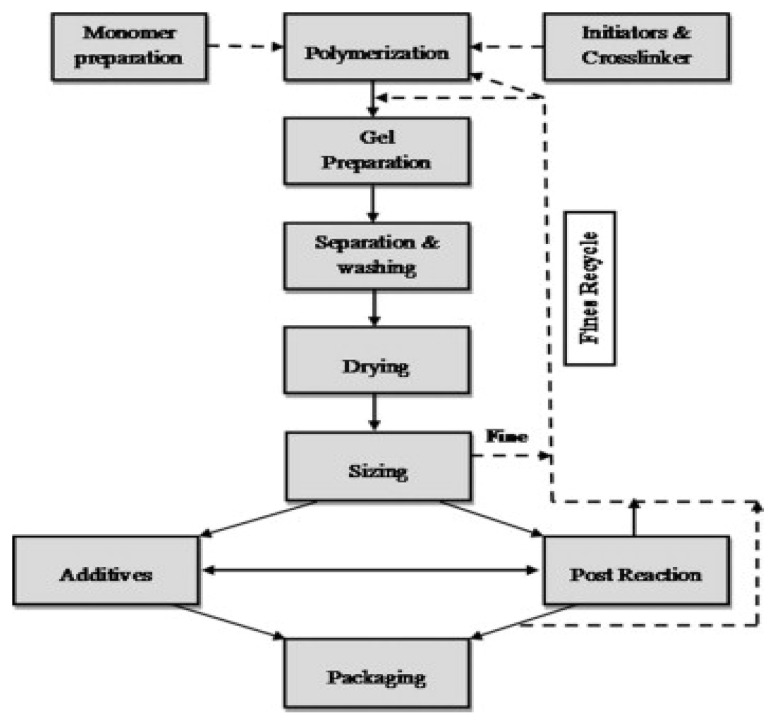
The primary steps for making hydrogels on a semi-pilot and industrial scale. Adapted from Ahmed et al. [76].

On the other hand, hydrogel materials are frequently made by polymerizing acrylic monomers using a free radical initiator [76]. The resins can be made in an aqueous medium with solution polymerization or in a hydrocarbon medium with well-dispersed monomers (Figure 5 and Figure 6). Hydrogel has gained a lot of attention in recent years due to its exceptional mechanical properties, swellability, biocompatibility, etc., but there are still a few things that can be done to improve it. A simple approach was reportedly used to make a novel alginate/graphene double-network (GAD) hydrogel, and its mechanical characteristics, stability, and adsorption performance were compared with alginate/graphene single-network hydrogel (GAS) [77]. It was discovered that GAD has a smaller swelling power than GAS, resulting in better gel stability in highly concentrated alkali/salt solution. The GAD beads have a considerably greater adsorption capacity for heavy metal ions and dye than GAS beads.

Apart from the adsorption of aqueous- and gas-phase pollutants, several aerogel composites are acknowledged as useful tools for photocatalytic remediation by harnessing the (photo)catalytic moieties present in their framework [78]. Although aerogels and hydrogels are good remedial tools, there is still the challenge of sophisticated drying technology requirements, structural fragility and instability, and high processing costs, which should be subject to advanced research [78,79]. To strengthen the structure of aerogels and hydrogels, for improved performance and wider applications, aerogel and hydrogel-based nanocomposites have been developed (Table 2).

**Figure 7:**
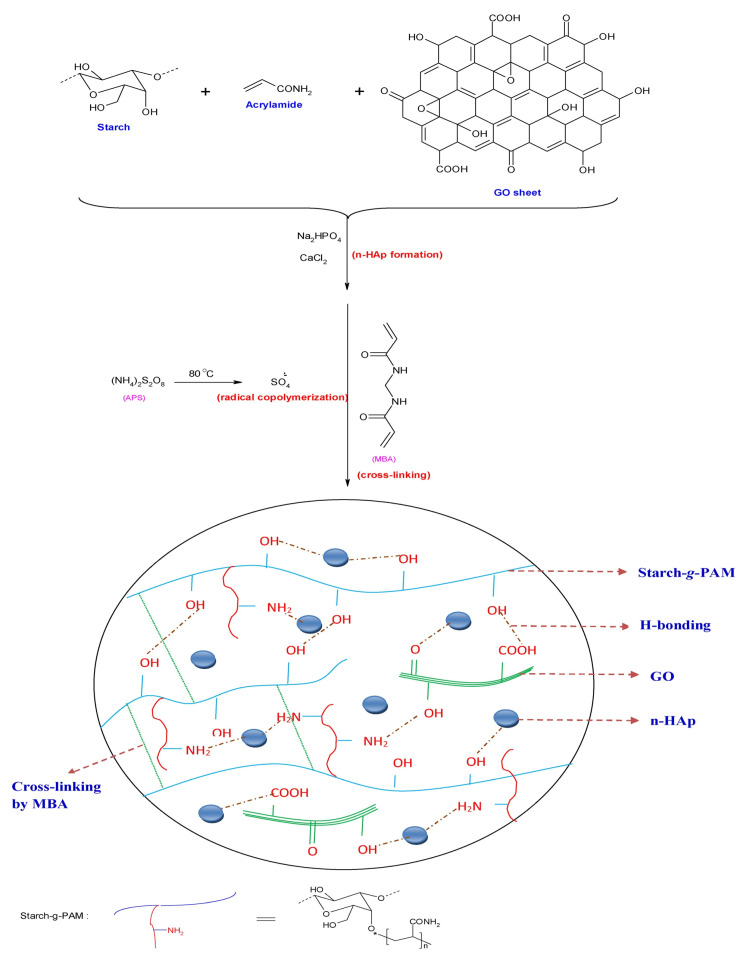
Hydrogel adsorbent with starch-graft-poly(acrylamide)/graphene oxide/hydroxyapatite nanocomposite as constituent. Adapted from Hosseinzadeh and Ramin [80].

A starch-graft-poly(acrylamide)/graphene oxide/hydroxyapatite nanocomposite hydrogel was prepared by free radical cross-linking copolymerization [80]. It is worth noting that the n-HAp nanoparticles served as cross-linkers, allowing for ionic interactions and hydrogen bond formation between phosphates and the polymer network’s grafted acrylic groups. It also demonstrated that many hydrogen bonds and potential covalent connections developed between electrons of graphene oxide (GO) sheets and acrylamide (AM) monomers grafted on a starch framework (Figure 7). The bioadsorbent was used for malachite green dye adsorption. The results revealed that the adsorptive interaction was viable, spontaneous, and endothermic. The pseudo-second-order model was used to characterize the malachite green (MG) sorption rates. The sorption data were best-fit by the Langmuir model with a maximum adsorption capacity of 297 mg/g. The hydrogel-based adsorbent demonstrated good regeneration capacity for up to five cycles [80]. The polymeric nanocomposite could be an environmentally benign and promising adsorbent for water treatment applications.

Similarly, a starch/MnO_2_/cotton hydrogel nanocomposite has been synthesized [81]. 0.008 M potassium permanganate, 0.7 g starch, and 0.6 M sodium hydroxide were used to make the optimal starch hydrogel nanocomposite at 50–55 °C. Potassium permanganate was used as a strong and cheap oxidizing agent to prepare manganese dioxide nanoparticles and cross-link the starch molecular chains to cellulose molecular chains. Because of its simple one-step synthesis approach, in-situ preparation of nanoparticles, cost-effectiveness, and desirable activity such as photocatalysis, biocompatibility, antibacterial properties of 93 percent against *S. aureus*, the starch hydrogel nanocomposite is a good material for various applications such as agriculture, medical, textile engineering, and water treatment [81].

A selective adsorbent was developed for the remediation of mercury (Hg) contaminants in aqueous solution [82]. The functionalized silica-gelatin hybrid aerogel with 24 wt.% gelatin content is ideal for the high efficiency and selective adsorption of aqueous Hg(II). By exposing cultures of *Paramecium caudatum* to Hg(II) and observing the model cultures with time-lapse video microscopy, the remediation efficacy of this adsorbent was assessed under realistic aquatic settings. With Hg(II) concentration, the viability of *Paramecium* shows a definite exposure-response relationship. When the Hg(II) concentration is greater than 125 g/L, viability diminishes. Only at Hg(II) concentrations greater than 500 ppm do the cells lose viability in the presence of 0.1 mg/mL aerogel adsorbent. The importance of the provided quasi-realistic aquatic toxicity model system in meeting the needs of environmental and chemical engineering technology is highlighted by the need for actual testing during adsorbent development [82].

**Table 2:** Selected hydrogel/aerogel nanocomposites for water treatment applications.

Material Description	Core Findings	Reference
MnO_2_ coated cellulose nanofibers	Oxidation occurred at acidic pH. Over 99.8% removal of methylene blue dye	[83]
MnO_2_/graphene aerogel (GMA)	GMA had 100% adsorption of rhodamine B and 89.02% COD, compared to 73.80% and 59.65% for SMA (silica wool-MnO_2_ deposition)	[81]
Poly(acrylic acid)/starch hydrogel	Adsorption of cadmium was best described by Langmuir (monolayer) adsorption model with a maximum adsorption capacity of 588 mg/g	[84]
3D MnO_2_ modified biochar-based porous hydrogels	Cd(II) and Pb(II) removal from aquatic and soil systems could be possible uses. Reusable and highly stable	[85]
Cassava starch-based double network hydrogel	The high adsorption capacity of about 417 mg/g and adsorption performance of 70% after regeneration five times. Physically and mechanically stable.	[86]
Chitosan-Gelatin based hydrogel	CH-GEL/ZSPNC (MW) eliminated 99% of cationic dye from the solution. The adsorption capacity of about 10.5 mg/g	[87]
CdS amended nano-ZnO/chitosan hydrogel	For 5.0 mg/L, 95 percent of Congo Red was removed in 1 min. Pollutant removal is quick, with high apparent rate constants and good reusability.	[88]
MnO_2_ NWs/chitosan hydrogels	Abundant sunlight absorption (94%). The conversion efficiency of sunlight to thermal energy (90.6%)	[89]

### 2.3. Polymeric Membrane and Biopolymers

The need for freshwater will continue to rise over time, necessitating the development of cost-effective, eco-friendly water treatment technology [90,91]. Membrane processes such as reverse osmosis, membrane distillation, pervaporation, and others are among the existing technologies for drinking water management [10,92]. The mechanical and thermal stability of the present polymer membranes employed in water treatment procedures has various drawbacks [93].

Simple and cost-effective water filtration technologies based on recyclable biobased natural polymers such as chitosan, cellulose, and carbohydrate polymer modified with nanoparticles are needed [94]. These novel chitosan-based nanomaterials have been shown to effectively remove a range of pollutants from wastewater to permissible levels [95]. Other biobased polymer and polymeric support includes porous resins, polyaniline, polyacrylamides, cellulose acetate, cellulose or carboxymethyl cellulose, chitosan, alginate, eggshells, nanofibers and cellulose nanofillers (CNFs) [19,96]. CNFs potentially hold an important role in the advancement of the development of polymer nanocomposites. However, the challenge of extracting, isolating, and refining them, as well as surface improvements, can be developed systematically. It is safe to assume that CNF exploits will first find use in high-end applications, since their utilization offers significant benefits in limited quantities.

Membrane-based separation technologies are regarded as the most advanced separation technology due to various merits: they can meet a wide range of separation requirements; change of phase is not required, which saves energy; they are highly selective; they can retrieve trace amounts of contaminant; and they are simple, adaptable, and versatile [91,97]. Cellulose nanofillers have found applications in membranes/filters, injury dressings, dental implants, sophisticated adhesives, and materials requiring high transparency and mechanical qualities [98]. Findings suggest that when the choice of the polymer matrix is properly done, this enhances mechanical properties and lead to a high degree of optical transparency in the finished product [99]. The greatest barrier to their industrial applications remains the efficiency of recovery and facile strategies for morphological modifications as integrated with a system. It is worth noting that some recent research suggests that CNFs have increased barrier qualities as a function of cellulose crystal content, implying that they could potentially be used in more environmentally friendly packaging materials [100,101].

**Figure 8:**
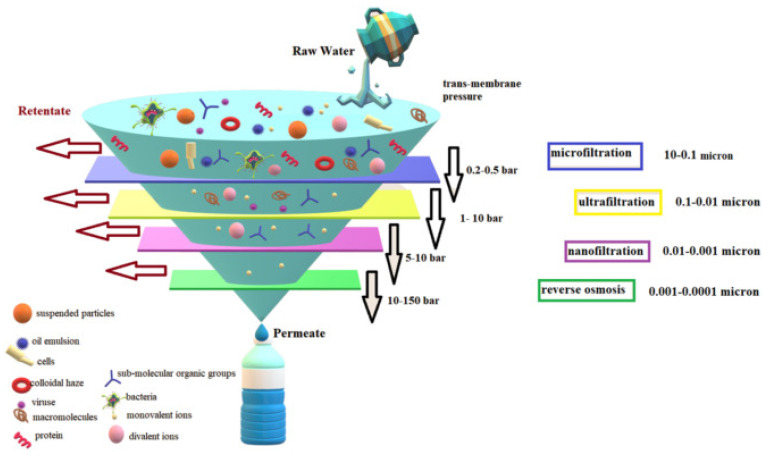
Capabilities of different separation/adsorptive membranes using microfiltration (MF), ultrafiltration (UF), nanofiltration (NF) and reverse osmosis (RO) processes. Adapted with permission from Hmtshirazi et al. [19]. Copyright (2022) Elsevier.

Adsorption and rejection are the two strategies for extracting solutes from wastewater using adsorptive membranes. Molecular sieving and filtration rejects any solutes larger than the membrane’s pore size when water-containing solutes come into contact with the active layer of the membrane (Figure 8). Smaller solutes will pass through the active layer and into the support layer, which acts as an adsorption microsphere [19,102]. These solutes then react/bind to form a tight internal spherical complex, resulting in filtered water permeating from the adsorptive membrane that meets the requisite standards [103]. The nanofiltration (NF) and reverse osmosis (RO) membranes are widely utilized due to their high-water permeability, low-pressure need, and low cost (Table 3). The improvement of the membrane’s functional adsorption sites has been reported to enhance its performance [104]. Therefore, using hydrophilic nanoparticles to boost the clean treated water flux can be quite successful, as long as the number of nanoparticles introduced is low. Having less than 6% adsorbent in the membrane matrix is typically recommended to avoid clogging membranes and counterproductive effects [105,106].

Recently, a carbon molecular sieve (CMS) with different concentrations was used to make asymmetric polyethersulfone (PES-15 wt. percent) mixed-matrix membranes via the phase inversion method [107]. Loading 1 wt.% of CMS, the membranes improved in hydrophilicity, heterogeneity, porosity, net surface charge and mean pore diameter. Furthermore, 1 wt.% CMS addition to PES, led to increased pure water flux from 55.77 to 75.05 L/m^2^.hr. This polymeric nanocomposite showed improved dissolved ions-removal efficiency, compared to the unmodified PES membrane [107]. Table 3 presents various polymeric membranes that have been used to remove several types of pollutants from water, and the process parameters as well as the treatment technology adopted by various researchers.

**Figure 9:**
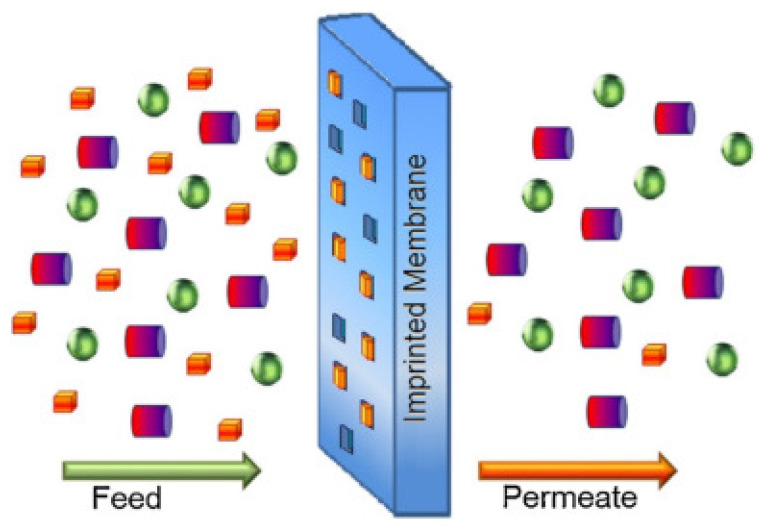
Removal of multiple contaminants selectively via an adsorptive membrane. Adapted from Salehi et al. [106].

Polymeric adsorptive membranes are generally considered potent pollution remediation technologies and have various applications in wastewater treatment plants and household water polishing. Cellulosic and other polymeric membranes have the capacity to remove various classes of persistent and emerging chemical pollutants from wastewater that are recalcitrant to conventional methods. The combined advantage of adsorption and filtration mechanisms, as well as the variety of forms and configurations, make these imprinted membranes (or membranes in general) and biopolymers appealing (Figure 9). Adsorptive membranes address fouling, minimize operational cost, adsorbent reusability, and enhances adsorption capacity, membrane permeability, rejection rates, and selectivity.

**Table 3:** Selected polymeric membrane nanocomposites for water treatment applications.

Polymeric Membrane	Treatment Technology	Target Pollutants	Core Process Conditions	Reference
ES-10- polyamide, NTR-729HF- polyvinyl alcohol	Reverse osmosis (RO)	As, Sb	As(V) and Sb(V) removals are substantially higher than As(III) and Sb(V) removals at pH 3–10.	[108]
ES-10 and HS5110/HR3155	Nanofiltration (NF)/RO	As	NF: pressure 0.2–0.7 MPa/RO: pressure 4 MPa	[109]
NF90–4040	NF	Cr, As	pH = 9, temp. 45 °C, pressure 3.1 MPa	[110]
UiO-66 (Zr-MOF)/TFN	NF	Se, As	1.15 L/m^2^·h/MPa	[111]
The P[MPC-co-AEMA] co-polymer	NF	Se, As	0.85 L/m^2^·h/MPa	[112]
PVDF with melanin nanoparticles from the marine bacterium *Pseudomonas stutzeri*	Vacuum filtration (VF)	Hg, Cu, Cr, Pb	45 °C; pH = 3 for Cr and pH = 5 for other metals; flow rate of 0.5 mL/min	[113]
M-I	Micellar enhanced filtration (MEF)	Cu, Pb, Cd	Operating pressure 0.025 MPa; the flux 63,579 L/m^2^ h	[114]
PAN- Polyacrylonitrile—Osmonic 100 kDa	Electro-ultrafiltration (EUF)	As	an averaged crossflow velocity of 0.1 m/s; pressure 0.098 MPa	[115]
Desal AG-2540 RO, TFC-ULP-2540 RO, and TFC-SR2-2540 NF	NF/RO	Sr	Applied pressure 0.10–0.15 MPa, pH = 3–6	[116]
Polyelectrolyte multilayer membrane	NF	Mg, Sr, Ca, Ba	low ionic strength conditions (e.g., <50 mM NaCl as a background electrolyte); 0.345 MPa; crossflow velocity 21.4 cm/s; 25 °C.	[117]
tubular Kerasep^®^ ceramic membranę	Hybrid: Oxidation	Fe	Oxidation: 0.07 MPa; 20–22 °C; MF: tangential velocity 3.2 m/s; trans-membrane pressure 0.06–0.3 MPa; pH = 6.8–7.2; 20–22 °C	[118]
PPSU—sulfonated polyphenylenesulfone polymer; TBF—triangle-shape tri-bore hollow fiber membranes	UF	Oil	Transmembrane pressure of 0.1 MPa; a flow rate of 300 mL/min along the lumen side; a velocity range of 2.58–2.81 m/s	[119]
NiCo-LDH—nickel cobalt layered double hydroxide; PVDF—the polydopamine modified polyvinylidenefluoride membrane	Gravity-driven filtration	Soybean oil, petroleum ether, 1,2-dichloroethane, n-hexadecane	Glass sand core filter device; water-in-oil emulsions—the volume ratio of 1:99	[120]
APTES—3-aminopropyltriethoxysilane; ATPR—atomic transfer radical polymerization/Graphene oxide	Filtration	Oil	Polymerization with ATRP; a volume ratio of organics and water: 1:99; the pressure of 0.05 MPa; complex environments, such as 2 M HCl, 2 M NaOH and saturated NaCl; permeation flux 10,000 ± 440 L/m^2^·h·MPa	[121]
Nanofibrous PVDF membrane	Gravity-driven filtration	Oil	Permeability 88 1660 ± 6520 L/m^2^·h·MPa; water-in-oil emulsions (chloroform, toluene, dichloromethane, and high viscosity oils: D4 and D5)	[122]
TiO_2_-Nanoparticles/PVDF—polydopamine modified polyvinylidenefluoride membrane/TrFE—trifluoro ethylene	Photoreactor	Oily industrial wastewater	The flow rate 100.8 L/h; pH = 4–5.5	[123]
SiO_2_-NPs/PVDF	Separation	Oil	The pressure of 0.09 MPa; fluxes of over 10,000 L/m^2^ h	[124]
PVDF—polydopamine modified polyvinylidenefluoride membrane	RO	Oil	The cross-flow velocity 2 m/s; operating pressure 6 MPa; crossflow membrane sequencing batch reactor inoculated with isolated tropical halophilic microorganisms	[125]
Chitosan–SiO_2_–glutaraldehyde composite/PVDF- polydopamine modified polyvinylidenefluoride membrane	VDF system	Oil	Separation area ~1.6 cm^2^; the pressure 0.03 MPa.	[126]
TiO_2_-NP/polydopamine modified polyvinylidenefluoride membrane	Separation	Petroleum ether; n-hexadecane; 1,3,5-trimethylbenzene; diesel oil	Pressure difference of 0.09 MPa; separation area 1.77 cm^2^; permeation flux for SDS/oil/H_2_O emulsion: 428 L/m^2^∙h, 605 L/m^2^∙h, 524 L/m^2^∙h, 382 L/m^2^∙h respectively	[127]

## 3. Considerations for Future Research

Pollutants in aqueous media have been remedied using a variety of polymeric nanocomposites adsorbents [128]. The toxicity of the adsorbent, when used for water filtration, is a major concern. Cellulose and other carbonaceous adsorbents (i.e., graphene, carbon nanotubes) have received significant attention for water purification due to their large surface area and affinity for inorganic and organic pollutants, as well as microbial contaminants [129,130]. Their toxicity, on the other hand, is relatively unknown and this requires more evaluation. Carbonaceous compounds and organic-based nanomaterials with reactive moieties may be hazardous to human health [131]. Therefore, only rational chemical design combined with a comprehensive understanding of potential biological interaction and risks can result in the fabrication of polymeric nanocomposites that is both safe and effective [18].

In recent research, polymers have been combined with materials such as carbon nanotubes, graphene oxide, and magnetic nanoparticles, in order to improve physicochemical and morphological properties [51,53]. Therefore, it makes sense to evaluate the toxicity of the entire composite system and not just the polymers. It is a prevalent fallacy that certain metals are only harmful in their pure form and functionalizing with a polymeric framework lessens toxicity. However, such statements should be backed up by scientific evidence.

Polymeric membrane technology has some concerns and challenges that need to be addressed by the scientific community. Examples include, but are not limited to:

(1) Fouling has long been a severe issue encountered during polymeric membrane applications in water treatment. Antifouling nanoparticles and surface functionalization are some of the ways to address this challenge (post or pre-treatment) [132]. Future studies should concentrate on inhibiting the growth of microbial colonies on the surface of the membrane, as well as minimizing filler leaching.
(2) In real-world applications, polymer nanocomposites’ availability, reusability, cost, stability, agglomeration, and reactivity are all major concerns. As a result, developing novel, inexpensive, and effective nanofillers and polymeric nanocomposites for adsorptive membrane technology still requires attention.
(3) It is difficult to ensure that the adsorptive material combined with the polymeric membrane is safe and harmless. Some composite materials are hazardous because their application in water purification generates secondary pollution. Environmental health and human safety can be achieved by carrying out comprehensive post-treatment evaluation to determine the quality of the water, its suitability for human consumption, and/or its safety for release into the water bodies.
(4) The development of new materials for polymeric nanocomposites remains a major issue, as most materials have been limited to laboratory-scale testing and advanced field trials are needed. Because many innovative materials are not marketable yet due to high pricing or time-consuming synthesis procedures. There is a need for continuous material science research for sustainable and cost-effective polymeric membranes.
(5) In addition to identifying the necessary steps for scaling up new membranes for large-scale industrial applications, there is a need for the development of facile synthesis methods capable of producing defect-free polymeric membranes, without compromising water treatment efficiency.
(6) Furthermore, models for the prediction of the lifespan of the polymeric membrane, regenerability, and reusability are required. To forecast membrane performance and economic viability, models that take into account the morphology and specific characteristics of the polymeric nanocomposite must be developed and validated.

## 4. Conclusions

Polymer nanocomposites’ development and application in water and wastewater treatment holds immense potential due to the added benefits of the synergism between adsorption and membrane filtration approaches. Polymeric membranes have shown remarkable performance in removing both established and emerging chemical pollutants from water. In the synthesis of polymer nanocomposites, water, atomic layer deposition, and plasma-assisted mechanochemistry have been identified to solve the problem of nanoparticle aggregation in melt-mixing techniques. Mixing techniques also benefit from sonication at high frequencies. In-situ polymerization allows for the creation of thermodynamically stable polymeric nanocomposites. Electrospinning is an efficient way for developing porous materials. In addition, fabricating nanocomposites with selective laser sintering offers obvious advantages in terms of avoiding aggregation.

Furthermore, the diversity in the types of materials, i.e., dendritic, biopolymeric, cellulosic and various polymeric composites, available for adsorptive membranes application is advantageous. However, the use of polymeric nanocomposites as adsorbents or as membranes (involving both filtration and adsorption) have various challenges associated with their preparation, human and environmental health, and as well as scalability and adaptability for industrial and field applications. As a result, future research should focus on overcoming these obstacles in order to effectively and sustainably use polymeric nanocomposite adsorbents and membranes in water treatment sectors.
